# Transactions of the Chicago Society of Ophthalmology and Otology

**Published:** 1885-11

**Authors:** Boerne Bettman

**Affiliations:** Secretary; 113 Adams street


### SOCIETY REPORTS.

---

### TRANSACTIONS OF THE CHICAGO SOCIETY OF OPHTHALMOLOGY AND OTOLOGY.

The Chicago Society of Ophthalmology and Otology met August 11. Drs. F. C. Green and F. W. Coleman were proposed for membership. The following papers were then read:

One on an

### OPERATION FOR MALFORMED AURICLE, by Dr. S. S. BISHOP.

He said: On July 11th, Nettie H., eight years old, presented herself at my clinic at the Illinois Charitable Eye and Ear Infirmary, and said she "wanted her ears put back." Both auricles were so abnormally prominent as to mar materially the symmetry of a very shapely face and head. The left auricle, which was the more unsightly of the two, projected one and a quarter inches at a right angle from the junction of the auricle with the mastoid process, and then bent forward, thus forming what is facetiously termed "lop ear," or "dog ear." The natural elevations and depressions were obliterated, and the organ presented the appearance of having been flattened by the pressure of the head in sleep on an auricle folded forward upon itself. The right ear projected forward one inch, but was not so imperfectly shaped as the left one.

At first I contemplated moulding the auricle into shape in a dressing of plaster of Paris, which would retain the parts in position for several months, with proper care and renewed dressings. But on account of the youthfulness of the child, which would render it highly improbable that she would co-operate in that treatment with sufficient persistence to insure success, I performed an operation, with the consent of her parents, and the assistance of Drs. Hawley, Abbott and Walker.

On July 17th last, the little patient was anæstethized with ether. An incision one and three-quarters inches long was made on the posterior surface of the auricle, about one-half inch from the free border of the cartilage and parallel therewith. Another curvilinear incision internal to the first was made, to unite its extremities. Those incisions extended only through the skin and subcutaneous tissue, and were made to embrace about one-half inch of integument at the widest divergence of the lines of incision. After dissecting off this skin and subcutaneous tissue, two incisions parallel to the first were made through the cartilage, and an elliptical section of this was dissected off from the skin covering the anterior aspect of the auricle without wounding that integument. The portion of cartilage removed was not quite as wide or long as the section of skin taken from the posterior surface of the auricle. The edges of the wound were united by three sutures, which included the skin only, and the parts were dressed with absorbent cotton and wet bandage. Union by first intention occurrred, and the sutures were removed the fifth day. However, the patient chose a pleasure excursion in preference to the clinics before the wound had healed thoroughly, the dressing became displaced and the wound was torn open along the center. Consequently this portion of the wound healed later by granulation. There was but slight pain or swelling, and the child evinced no dread of an operation on the other ear, except with respect to the ether.

The result of the operation is that the auricle now projects but three-fourths of an inch instead of one and a quarter inches, making a difference of one-half inch between the projection before and after the operation. Besides this, the natural elevations and depressions which are requsite to the beauty of a well-formed ear have been restored.

I do not know that any surgeon has previously performed an operation identical with the one I have devised, but when a learned professor informs the French Academy of Sciences that before the illustrious Jenner was born, and "from a period so remote that it loses itself in the night of time," the inhabitants of Senegambia have practiced inoculation for the prevention of a contagious disease, I have not the temerity to call any operation new. A short time since I thought I had originated another new method of treatment, when, to my surprise, I learned that Dr. Sexton, of New York, had been experimenting in the same direction, although he had not published the fact.

Drs. Ely and Roosa have operated in a different manner to effect a similar result. They transfixed the auricle at its junction with the side of the head and removed the cartilage and the skin covering both the anterior and posterior surfaces. By the operation which I performed it is apparent that the integument covering the anterior surface of the auricle, or the part most exposed to view, was not at all injured. I was not able to ascertain the exact result in Dr. Ely's case. In Dr. Roosa's case the projection before the operation was the same as in mine, but the projection in my case after the operation was one-eighth of an inch less than in his case.

The next paper, on

SUN-BLINDNESS, was read by Dr. C. F. SINCLAIR.

He said: The danger from blindness from the direct rays of the sun, which appears to have been generally recognized, not only by the medical profession, but by the laity as well, of the past generation, is apparently unrecognized to-day. During the recent eclipse many might have been seen looking at it with the naked eye. It is not uncommon by patients to tell the physician, as a proof of strong vision, that they can look steadily at bright lights, and even at the sun, without blinking. Children not unfrequently undertake competitive trials to see which can gaze longest and most steadily at the sun.

In many of the recent smaller works on ophthalmology the subject of sun-blindness is not mentioned.

Nettleship, Lawson, Williams, Schiœps and DeWecker refer to the possibility of blindness being caused by flashes of lightning, by the bright glare from snow, and from a too-prolonged gaze at a brilliant artificial light, but the possibility of a like result following from the direct rays of the sun is not referred to. Sœlberg Wells mentions the subject, but in the most cursory manner, simply saying that blindness may follow if the eye is long exposed to bright sunlight. He further points out the difference which may exist in the density of the cloud or disk, and which may last only a few moments, or days, or weeks, or even longer. The prognosis in his view appears to be favorable. No reference is made to any pathological condition. The treatment recommended is rest, protection from bright light with suitable colored glasses, cod-liver oil and steel. The majority of recent writers, however, have ignored this as a cause of blindness—a cause which, from the carelessness of adults and the ignorance of children, may well be considered one which is most practical and vital.

In a more recent work by Swanzy, of Dublin, the subject is considered at somewhat greater length. He refers to the semi-blind spot which appears in the field of vision after exposure, and which, however, may be absolute. In his experience, even in mild cases, this cloud seldom entirely disappears. The ophthalmoscope shows a small bright white spot at the fovea centralis surrounded by a blood-red ring which fades off into the normal color.

A recent monograph by Deutchmann, of Göttingen, gives us more definite information as to the pathological conditions in these cases. His investigations were undertaken more particularly with that end in view. He cites four cases which came under his care in 1882. In one of these a blue glass had been used in looking at the eclipse. Still the result was the same. In all the cases, immediately after looking at the sun a small cloud appeared in the center of the field of vision, and examination showed a small central scotoma.

Vision was impaired in the same degree in three of the cases, being $\frac{20}{50}$. In one of the cases which came under observation two months after the first appearance of the blind spot, vision had already considerably improved, being $\frac{20}{30}$ without difficulty. The finest print in all these cases was read with difficulty. In none of Deutchmann's cases were the scotomata complete, and yet in none was perfect vision recovered. Pathological changes were found in all, viz., a small bright white spot in the centre of the macula lutea, and around this a red ring which gradually faded off into the normal retina. To verify these results Deutchmann undertook a series of experiments on the eyes of rabbits.

The direct rays of the sun were condensed by a concave mirror and then rendered parallel by a convex lens. They were then directed into the retina through the dilated pupil.

The resulting appearance was similar to those seen in the eye, *i. e.*, the small central spot of anæmia with the surrounding areola of hyperæmia—these results following an exposure of only a few seconds' duration.

An examination with the microscope showed that an extremely small area of the retina was disorganized by the coagulation of the albumen in its tissues with hyperæmia, exudation, diapedesis of blood corpuscles and pigment disturbance surrounding it.

Based upon these pathological changes, the unfavorable prognosis which Deutchmann gives in these cases would seem to be well justified. The following case, however, which came under my observation the 28th of last March, presents phenomena difficult of explanation, if we accept the view that these pathological changes are an essential element in sun-blindness.

Howard D., a lad thirteen years of age, looked at the sun several times during the eclipse of last March. Finally, after a somewhat prolonged gaze, he felt slightly dazzled. Then, wherever he looked, objects seemed colored with quickly alternated shades of green and blue. This lasted half an hour and disappeared. His vision continued good until 4 P. M. of the same day, when, looking at the snow or sky, he noticed a dark spot in the center of the visual field.

The spot was quite dark and about eight inches in diameter It was round, with a piece taken off the outer side—this piece representing the position of the moon when the eclipse was last observed. It appeared as if the sun had photographed itself upon the retina.

Three days later a dull ache was felt in and around the eyes.

I first saw the patient twelve days after the accident had occurred. He then told me that the cloud had been constantly growing larger and darker. Vision on this first examination was $\frac{7}{10}$ in both eyes.

The ophthalmoscope showed no discoverable morbid change in the fundus. The disk, the vessels and the retina all appeared in normal condition.

Subcutaneous injections of strychnine, together with the use of the constant current were begun. The beneficial effects of this treatment were seen four days later, when the cloud had become less dark and impenetrable, and the outline of letters representing normal vision or $\frac{10}{10}$ could be made out within its area. Still four days later the patient returned to school and attempted to go on with his studies. This resulted in a relapse of $\frac{7}{10}$ vision and an increase of size and density of the section, with a return of the dull aching pain in the eye. April 9, $\frac{10}{10}$ vision was again reached, but the letters could only be deciphered separately after closing and resting the eyes.

April 14th $\frac{10}{11}$ could be seen clearly for three seconds.

By April 21st, in the right eye twelve seconds were counted before the cloud came, and even then $_{1}\underline{_{0}}$ could be read through it. In the left eye six seconds elapsed before the cloud developed and through it $\frac{10}{10}$ could be seen only indistinctly.

April 28th, a week later, twenty seconds in the right eye and ten seconds in the left indicated continued improvement.

May 4th, the strength of the retina had increased to twenty-eight seconds in the right eye, and at the end of that time what the patient described as a simple blur came and $\frac{10}{10}$ could be easily read through it.

May 13th, the right eye had reached forty-three seconds and the left eye twenty-five seconds and only the upper half of the test types were obscure.

At this time, owing to considerable swelling and soreness over the temples, the injections of strychnia were suspended, when the strength of the affected portions of the retina were rapidly deteriorated to thirty-two seconds in the right eye and fifteen seconds in the left. The subcutaneous injections were again commenced, with an improvement in four days of eleven seconds in the right eye and twenty-two seconds in the left. This improvement was gradual until June 2d, when sixty-six seconds in the right eye and forty-six seconds in the left were counted before the development of the scotoma, which, at the same time, had diminished much in size and density.

At this time the patient withdrew from treatment.

The fact that this case, after several exposures, presented no discoverable pathological changes, together with the constant improvement, until sixty-six seconds were reached, during which no evidences of the scotoma were manifest, justify the conclusion that in these cases of sun-blindness the lesion may be much less serious than that indicated by Deutschmann—being rather of the nature of a paralysis than that of the disorganization of the retinal elements, and, consequently, that the prognosis as to the ultimate recovery of perfect vision is not necessarily improbable.

BOERNE BETTMAN,

113 Adams street. Secretary.

---

CHICAGO MEDICAL SOCIETY.

*Stated Meeting, Oct, 5, 1885*, the President, C. T. PARKES, M. D., in the chair.

